# DEVELOPMENT AND PILOT TESTING OF THE END-OF-LIFE READINESS ASSESSMENT (ERA) SURVEY

**DOI:** 10.1093/geroni/igz038.020

**Published:** 2019-11-08

**Authors:** Alexis M Coulourides Kogan, Julie Taguchi

**Affiliations:** 1 University of Southern California, Keck School of Medicine, Los Angeles, California, United States; 2 Ridley-Tree Cancer Center, Santa Barbara, California, United States

## Abstract

An emerging conceptual framework on the relationship between serious and sensitive end-of-life (EOL) discussions and patient hope points to the mediating role that patient readiness may play. Additional research has also found that among some patients, engaging in and EOL conversation before they are ready may actually cause harm. Presently, health practitioners do not have a way to measure patient readiness. Therefore, the purpose of this study was to develop a survey assessment tool, rooted in research, to measure patient readiness to engage in an EOL discussion. The 16-item survey was initially developed by a gerontological researcher and clinical oncologist, and tested for face reliability and appropriateness among a geriatrician and three patients with chronic illness. In May 2018, the final version of the survey was pilot tested among 168 patients attending their regularly scheduled oncology appointment. On average, most participants identified as Caucasian (74%) females (61%) in partnerships (58%) and having a cancer diagnosis (64%). Bivariate analyses revealed that older age (60+, p=0.008), Caucasian race (p=0.04), and reported greater knowledge of community and supportive services (p=0.049) was significantly associated with increased readiness to engage in an EOL discussion. Additionally, a higher score on the Advance Care Planning Engagement Survey was significantly associated with increased readiness (p<0.001). Findings reveal patient groups that might be more appropriate targets for interventions, education, and resources and highlight the role of interprofessionals in outpatient health settings to “prime the pump” for these types of conversations.

